# Benefits of an online multimodal nursing program among patients with peripherally inserted central catheter-related thrombosis

**DOI:** 10.3389/fpubh.2022.971363

**Published:** 2022-09-20

**Authors:** Xin Huang, Meilian Xie, Shan Zhao, Yingtong Chen, Liying Wu, Xiuqun Zeng

**Affiliations:** Department of Gynecology, Guangdong Provincial People's Hospital, Guangdong Academy of Medical Sciences, Guangzhou, China

**Keywords:** quality of life, psychological resilience, asymptomatic thrombosis, peripherally inserted central catheter, nursing

## Abstract

**Background:**

Asymptomatic peripherally inserted central catheter-related thrombosis (PICC-RT) is one of the most common and dangerous complications caused by peripherally inserted central catheter (PICC) insertion. A variety of factors might lead to huge psychological pressures on patients and markedly affect their quality of life. The aim of this study was to evaluate the benefits of an online multimodal nursing program on the quality of life and psychological resilience of asymptomatic PICC-RT patients with ovarian cancer.

**Methods:**

This was a prospective cohort study on patients with asymptomatic PICC-RT. Patients in the control group received routine nursing care, while patients in the intervention group obtained extra assistance through an online multimodal nursing program. Individual guidance, psychological support, and real-time consultation were provided to patients in the intervention group. All participants were followed for 3 months. The health-related quality of life and psychological resilience of patients were evaluated by using the 36-item Short Form Health Survey (SF-36) and Connor-Davidson Resilience Scale (CD-RISC), respectively.

**Results:**

Compared to baseline, most of the items in the SF-36 scores were significantly increased in both intervention and control groups after 3 months (all *p* < 0.05), except for the role emotional domain (*p* = 0.085 in control group). However, the SF-36 scores of the intervention group were significantly higher than those of the control group in All health domains, including physical functioning (*p* = 0.001), role physical (*p* = 0.004), bodily pain (*p* = 0.003), general health (*p* < 0.001), vitality (*p* < 0.001), social functioning (*p* < 0.001), role emotional (*p* = 0.002), mental health (*p* < 0.001) and health transition (*p* < 0.001). For CD-RISC scores, the mean value of the control group was 42.03 ± 4.42 at baseline and increased to 50.36 ± 4.70 after 3 months (*p* < 0.001), while the intervention group was 40.00 ± 6.61 at baseline and increased to 65.12 ± 5.21 after 3 months (*p* < 0.001). Moreover, the CD-RISC score in the intervention group was significantly higher than that in the control group after 3 months (*p* < 0.001).

**Conclusion:**

The application of an online multimodal nursing program could significantly improve the health-related quality of life and psychological resilience of asymptomatic PICC-RT patients. These findings provide evidence to support the necessity of an online multimodal nursing program in routine long-term follow-up, especially in the era of COVID-19.

## Introduction

A peripherally inserted central catheter (PICC) is a thin and long floating catheter inserted into central venous circulation through the peripheral vein of the forearm (1). It is a durable and reliable venous access device, that is frequently used in patients requiring long-term intravenous chemotherapy or parenteral nutrition (2). PICC can avoid repeated venipuncture and effectively reduce the incidence of phlebitis and tissue necrosis (3, 4). Nonetheless, severe complications can be caused by PICC insertion, including deep vein thrombosis, catheter-related endothelial damage, bloodstream infection and skin injury (5, 6). Among these, peripherally inserted central catheter-related thrombosis (PICC-RT) is the most common and dangerous complication (7, 8). This might result in a series of adverse consequences and induce the interruption of chemotherapy. Detachment of thrombi may cause pulmonary embolism and ischemic stroke, which are the leading causes of disability and death (9).

PICC-RT is classified into symptomatic and asymptomatic PICC-RT based on the clinical symptoms. Patients with symptomatic PICC-RT always present edema, tenderness, and acroparesthesia of the upper limb. Prior reviews mostly focused on symptomatic thrombosis, while little research has been reported on asymptomatic PICC-RT. However, current evidence suggests that asymptomatic PICC-RT presents a significantly higher incidence than symptomatic PICC-RT (10–12). The lack of attention on asymptomatic PICC-RT may eventually evolve to symptomatic thrombosis, which may increase the occurrence of postthrombotic complications and the risk of death (13). In addition, a lack of professional medical education and understanding of disease pathogenesis may lead to a sense of powerlessness in patients with asymptomatic PICC-RT. Moreover, patients may limit the movement of the upper limb after diagnosing thrombosis. Limited mobility of upper limbs and the maintenance of PICC may bring great inconvenience and distress to the patients. All of these problems bring huge psychological burdens to patients and impact their quality of life (14). Thus, it is vital to provide long-term professional monitoring and management to asymptomatic PICC-RT patients in physical, psychological, and social aspects. Unfortunately, limited studies have been devoted to exploring this area. In this study, we aimed to evaluate the utility and efficacy of an online multimodal nursing program on the quality of life and psychological resilience in asymptomatic PICC-RT patients.

## Materials and methods

### Patients

This was a prospective cohort study, conducted in accordance with STROBE guidelines. Based on preliminary results, the difference in the main outcome, mental health scores, was 3.92, and the difference in CD-RISC scores was 7.18 between the two groups. With an alpha level of 5% and a power of 80%, the lowest required sample size of each group was 31.

Inpatients with newly diagnosed asymptomatic PICC-RT were recruited between January 1, 2021, and December 31, 2021, in Guangdong Provincial People's Hospital. The inclusion criteria were (1) age ≥18 years, (2) newly diagnosed ovarian cancers through immunohistochemistry, (3) received six cycles of chemotherapy through PICC, and (4) discovered PICC-RT by ultrasound. The exclusion criteria were (1) the presence of clinical symptoms, such as upper limb swelling, tenderness, acroparesthesia or dysfunction, (2) arteriovenous thrombosis or coagulation disorder before PICC insertion, and (3) psychiatric disorders or cognitive illnesses before PICC insertion.

Written informed consent was obtained from each participant prior to participating in this research. This study was approved by the Ethics Committee of Guangdong Provincial People's Hospital and was performed in accordance with the Code of Ethics of the World Medical Association (Declaration of Helsinki).

### Measures

Health-related quality-of-life was measured by the 36-item Short Form Health Survey (SF-36) (15). This is a patient-reported questionnaire that covers eight health domains: physical functioning (PF), role physical (RP), bodily pain (BP), general health (GH), vitality (VT), social functioning (SF), role emotional (RE), and mental health (MH). Different options for each item have different score weightings. The final score ranges from 0 (worst general health status) to 100 (best health status) for each domain, and higher scores indicate better quality of life. The SF-36 questionnaire also includes a global health transition (HT) question that investigates the variation in general health status compared with 1 year prior. The response options for this item have five categories, which are “much better”, “somewhat better”, “about the same”, “somewhat worse”, and “much worse”.

The psychological resilience of patients was assessed by the Connor-Davidson Resilience Scale (C Connor-Davidson Resilience Scale D-RISC) (16). Resilience was defined as the personal ability to thrive in the face of adversity. The CD-RISC is a self-administered scale comprised of 25 items that declare psychometric properties. The scale is used to evaluate the feelings of participants over the past month. Each item is rated on a 5-point scale ranging from 0 (not true at all) to 4 (true nearly all of the time). The total score ranges from 0 to 100, with higher scores reflecting greater resilience.

### Procedures

For all ovarian cancer patients undergoing chemotherapy, routine color Doppler ultrasound was performed every week after PICC placement. Once asymptomatic PICC-RT was discovered, the patients were evaluated according to the inclusion and exclusion criteria. All eligible participants were evaluated and intervened according to Table 1. The time for initial identification of thrombosis was regarded as the time of study entry (T0). The general information of all participants was investigated by questionnaires in Chinese, including marital status, educational level, occupation, histological type, clinical stage, lymph node metastasis, and cancer-associated thrombosis. The initial health-related quality of life and psychological resilience of patients were evaluated by SF-36 and CD-RISC questionnaires in Chinese, respectively.

**Table 1:** The procedures for the control and intervention groups.

**Group**	**Initial assessment (T0)**	**Interventions**	**Final assessment (T3)**
Control group	• General information • Health-related quality of life evaluated by SF-36 • Psychological resilience evaluated by CD-RISC	Routine nursing care • Catheter maintenance • Therapeutic anticoagulation • Health education • Dietary guidance	• Thrombosis status evaluated by color Doppler ultrasound • Health-related quality of life evaluated by SF-36 • Psychological resilience evaluated by CD-RISC
Intervention group	• General information • Health-related quality of life evaluated by SF-36 • Psychological resilience evaluated by CD-RISC	Routine nursing care • Catheter maintenance • Therapeutic anticoagulation • Health education • Dietary guidance One-on-one professional guidance • Weekly education about PICC maintenance and anticoagulant therapy • Individual recommendations on diet, exercise, sleep and daily behavior • Professional guidance for family members • Real-time consultation and guidance • Psychological counseling • Professional psychological care	• Thrombosis status evaluated by color Doppler ultrasound • Health-related quality of life evaluated by SF-36 • Psychological resilience evaluated by CD-RISC

Those patients in the control group received routine nursing care, including catheter maintenance, therapeutic anticoagulation, health education and dietary guidance. Ultrasound evaluation and coagulation monitoring were performed at regular intervals.

In addition to the conventional nursing mentioned above, the patients in the intervention group obtained one-on-one professional guidance through an online multimodal nursing program. The knowledge about PICC maintenance and anticoagulant therapy was presented every week as texts, videos and pictures. The recommendations on diet, exercise, sleep and daily behavior were designed according to each patient's lifestyle. Professional guidance on how to concern, support and help patients was introduced to family members. Close communication was established through the instant messaging program. The investigators could solve the patients' questions and corrected their improper procedures in time. The psychological counselors also communicated with the patients regularly. If professional psychological care was necessary, a psychiatrist or psychologist intervened.

All patients participating in this study were followed for 3 months and underwent the final assessments (T3). Color Doppler ultrasound was used to identify the status of thrombosis. The coagulation indices were evaluated, including D-dimer fibrinogen, prothrombin time, activated partial thromboplastin time, thrombin time and prothrombin time/international normalized ratio (PT-INR). Health-related quality of life and psychological resilience were assessed using the SF-36 and CD-RISC questionnaires, respectively. Two researchers (XH and MLX) were responsible for the data collection.

### Statistical analysis

All statistical analyses were performed by SPSS software version 25.0 (IBM Corporation; United States). The general characteristics of the participants were analyzed by Student's *t*-test or Pearson's χ2 test. The Mann-Whitney *U test* or Student's *t*-test was used to compare the expression of coagulation indices and the scores of SF-36 and CD-RISC between the psychological intervention group and the control group. The variation in SF-36 and CD-RISC scores between T0 and T3 was determined by the Wilcoxon signed-rank test or matched samples *t*-test. A threshold of *p* < 0.05 was considered statistically significant.

## Results

### General characteristics

**Figure 1:**
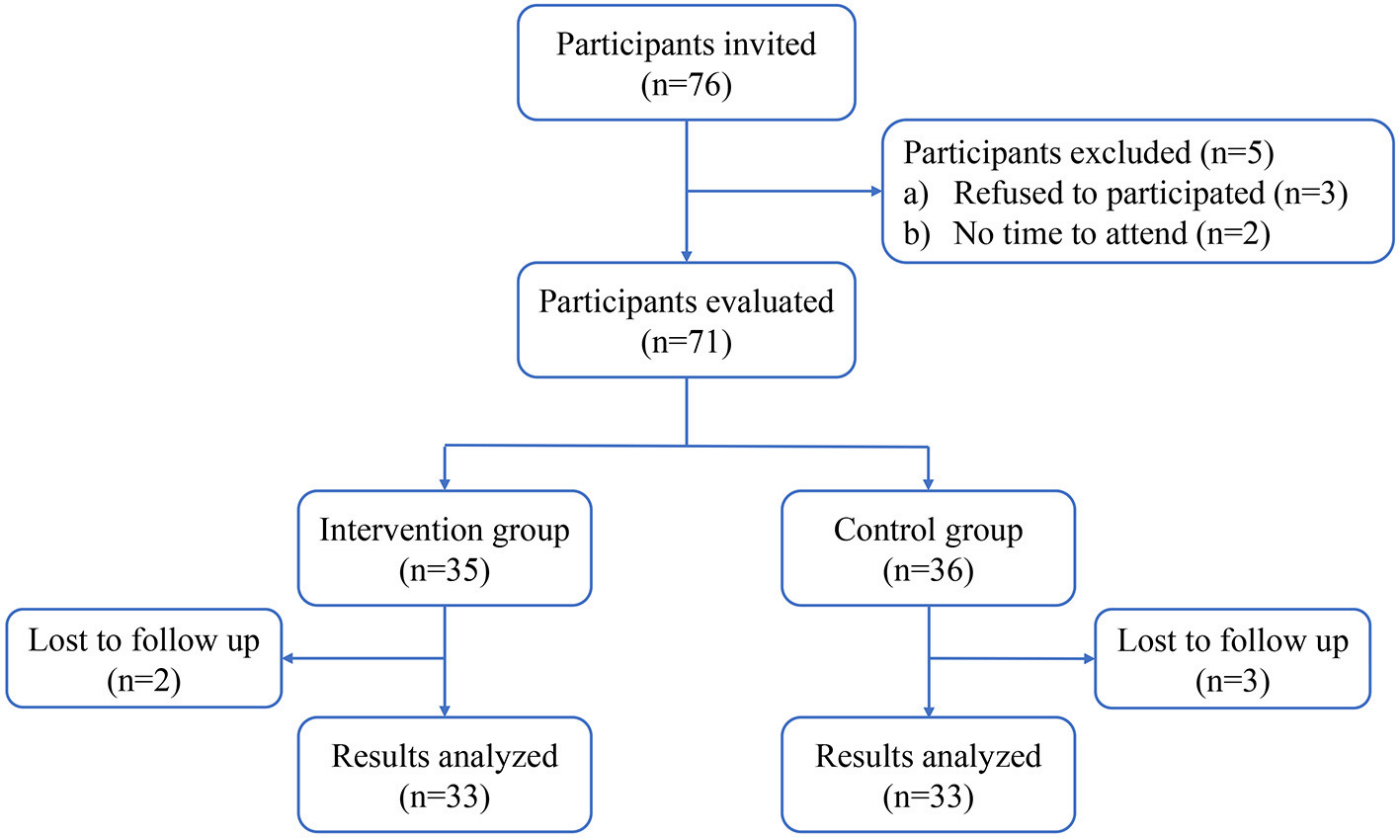
Flow diagram of the study.

The flow diagram of the participant selection process is shown in Figure 1. In total, 66 ovarian cancer patients with asymptomatic PICC-RT were enrolled in this study; half of the patients were in the intervention group, and half were in the control group. The general characteristics of the participants in the two groups are described in Table 2. There were no significant differences in patient age, body mass index (BMI), marital status, educational level, occupation, histological type, clinical stage, lymph node metastasis, or cancer associated thrombosis. The time from PICC insertion to thrombosis discovery was 28.33 ± 2.7 and 26.42 ± 2.48 days for the intervention group and control group, respectively. The difference was not statistically significant (*p* = 0.69).

**Table 2:** General characteristics of the intervention and control groups.

	**Intervention group** **(*n* = 33)**	**Control group** **(*n* = 33)**	***p* value**
**Age (y), M±SD**	50.85 ± 2.23	54.15 ± 2.03	0.648 ^a^
**BMI, M±SD**	21.29 ± 3.20	21.93 ± 3.27	0.785 ^a^
**Marital status**, ***n*** **(%)**			
Unmarried	6 (18.18%)	4 (12.12%)	0.625 ^b^
Married	24 (72.73%)	25 (75.76%)	
Divorced	2 (6.06%)	1 (3.03%)	
Widowed	1 (3.03%)	3 (9.09%)	
**Educational level**, ***n*** **(%)**			
High school or lower	22 (66.67%)	27 (75.51%)	0.159 ^b^
University degree or higher	11 (33.33%)	6 (24.49%)	
**Occupation**, ***n*** **(%)**			
Yes	17(51.52%)	14 (42.42%)	0.459 ^b^
No	16 (48.48%)	19 (57.58%)	
**Histological type**, ***n*** **(%)**			
Serous adenocarcinoma	24 (72.73%)	25 (75.76%)	0.767 ^b^
Mucinous adenocarcinoma	2 (6.06%)	1 (3.03%)	
Endometrioid adenocarcinoma	1 (3.03%)	2 (6.06%)	
Clear cell carcinoma	3 (9.09%)	1 (3.03%)	
Other	3 (9.09%)	4 (12.12%)	
**Clinical stage**, ***n*** **(%)**			
I	5 (15.15%)	8 (24.24%)	0.246 ^c^
II	1 (3.03%)	3 (9.09%)	
III	18 (54.55%)	15 (45.46%)	
IV	9 (27.27%)	7 (21.21%)	
**Lymph node metastasis**, ***n*** **(%)**			
Yes	13 (39.39%)	10 (30.30%)	0.438 ^b^
No	20 (60.61%)	23 (69.70%)	
**Cancer-associated thrombosis**, ***n*** **(%)**			
Yes	12 (36.36%)	6 (18.18%)	0.097 ^b^
No	21 (63.64%)	27 (81.82%)	
**Time of PICC-RT formation (*****d*****), M** **±SD**	28.33 ± 2.70	26.42 ± 2.48	0.690 ^a^

^a^Student's *t*-test; ^b^Pearson's χ2 test; ^c^Mann-Whitney *U* test; M ± SD, mean ± standard deviation; *BMI* body mass index.

**Figure 2:**
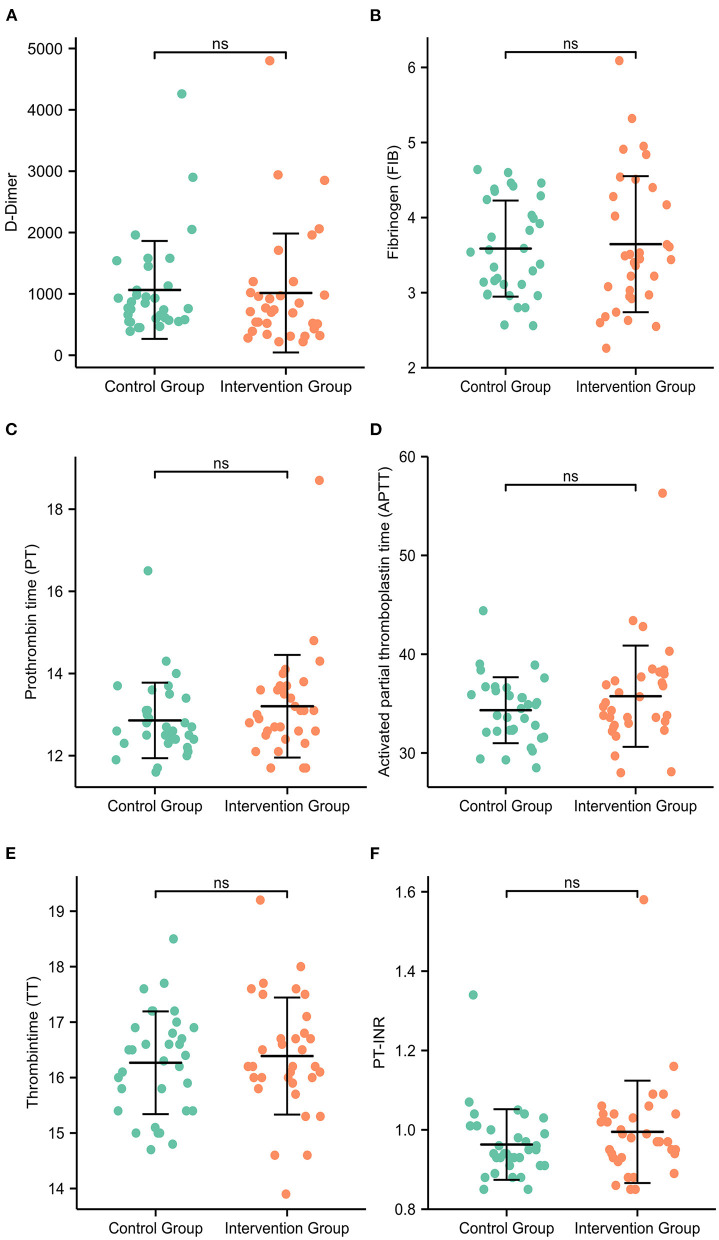
The coagulation indices of patients at the 3-month follow-up. There were no statistically significant differences in the expression levels of D-dimer **(A)**, fibrinogen **(B)**, prothrombin time **(C)**, activated partial thromboplastin time **(D)**, thrombin time **(E)** or PT-INR **(F)** between the intervention group and the control group.

After 3 months of anticoagulant therapy, the complete dissolution rates of thrombi were 96.97 and 93.94% in the intervention group and control group, respectively (*p* = 0.558). As shown in Figure 2, no statistically significant differences were found in the coagulation indices, including D-dimer (*p* = 0.243), fibrinogen (*p* = 0.964), prothrombin time (*p* = 0.113), activated partial thromboplastin time (*p* = 0.225), thrombin time (*p* = 0.621), and PT-INR (*p* = 0.171), between two groups.

### Health-related quality of life

**Figure 3:**
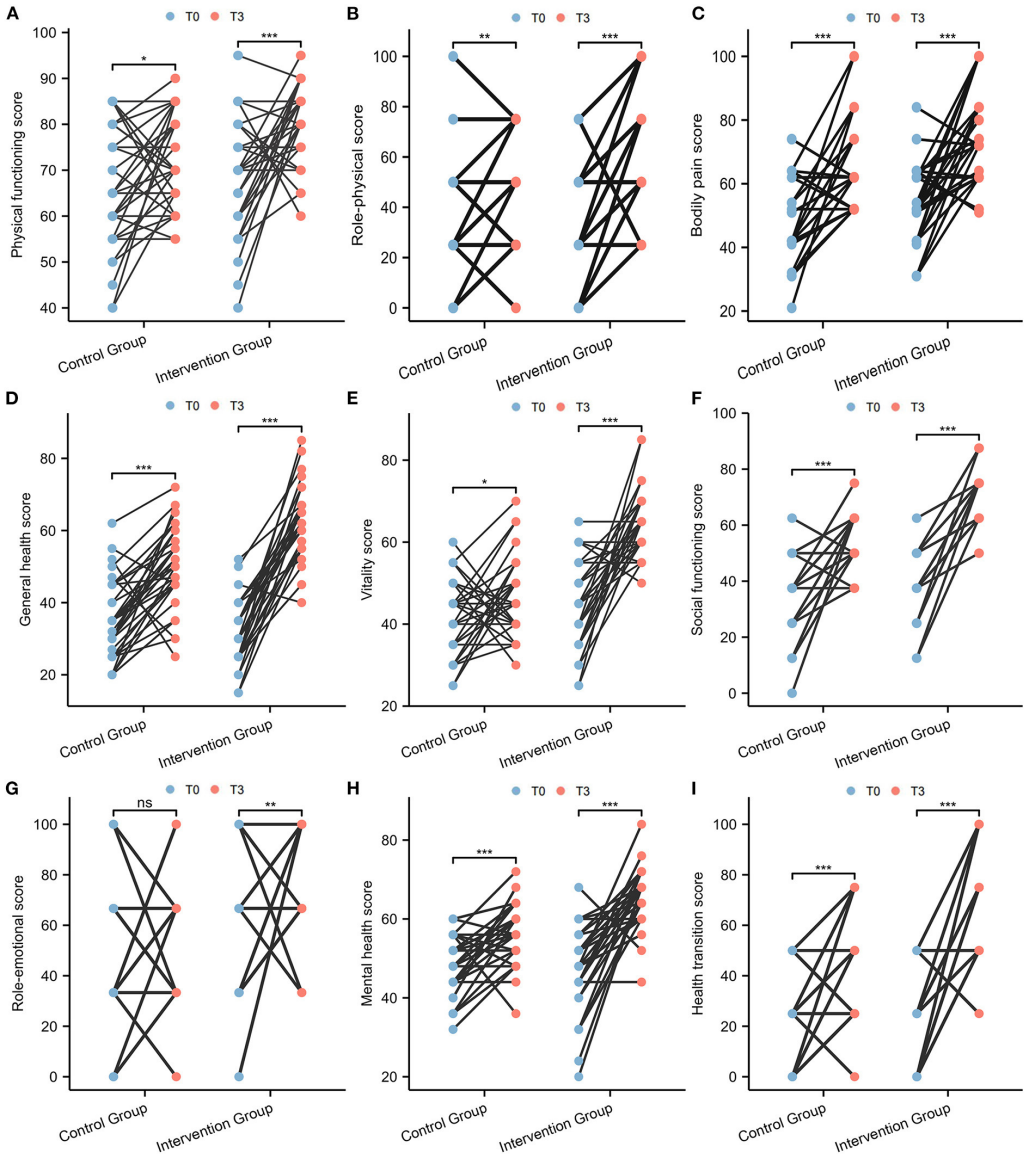
Comparison of SF-36 scale scores between T0 and T3 in paired samples. **(A)** Physical functioning; **(B)** Role physical; **(C)** Bodily pain; **(D)** General health; **(E)** Vitality; **(F)** Social functioning; **(G)** Role emotional; **(H)** Mental health; **(I)** Health transition.

**Figure 4:**
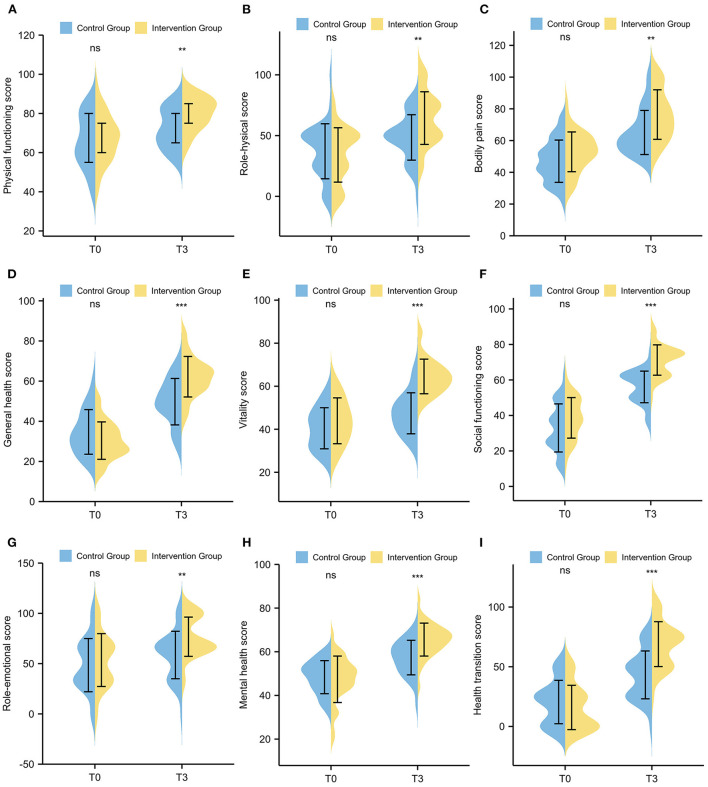
Comparison of SF-36 scale scores between the intervention group and the control group. **(A)** Physical functioning; **(B)** Role physical; **(C)** Bodily pain; **(D)** General health; **(E)** Vitality; **(F)** Social functioning; **(G)** Role emotional; **(H)** Mental health; **(I)** Health transition.

The SF-36 scores at baseline (T0) and three-month follow-up (T3) are shown in Table 3. Compared to baseline (T0), the intervention group had a significant increase in all health domains of the SF-36 scales after 3 months, including physical functioning (*p* < 0.001), role physical (*p* < 0.001), bodily pain (*p* < 0.001), general health (*p* < 0.001), vitality (*p* < 0.001), social functioning (*p* < 0.001), role emotional (*p* = 0.002), mental health (*p* < 0.001), and health transition (*p* < 0.001). For the control group, the scores of physical functioning (*p* = 0.038), role physical (*p* = 0.009), bodily pain (*p* < 0.001), general health (*p* < 0.001), vitality (*p* = 0.011), social functioning (*p* < 0.001), mental health (*p* < 0.001) and health transition (*p* < 0.001) were also significantly elevated after 3 months, except for the role emotional domain (*p* = 0.085) (Figure 3). However, the SF-36 scores of the intervention group were significantly higher than those of the control group in all health domains, including physical functioning (*p* = 0.001), role physical (*p* = 0.004), bodily pain (*p* = 0.003), general health (*p* < 0.001), vitality (*p* < 0.001), social functioning (*p* < 0.001), role emotional (*p* = 0.002), mental health (*p* < 0.001), and health transition (*p* < 0.001) at T3 (Figure 4).

**Table 3:** SF-36 and CD-RISC scores of the intervention and control groups at baseline (T0) and three-month follow-up (T3).

	**Intervention group**		***p* value**	**Control group**		***p* value**
	**T0**	**T3**		**T0**	**T3**	
Physical functioning score	67.42 ± 2.14	80.15 ± 1.48	**<0.001** ^a^	65.91 ± 2.36	71.97 ± 1.75	**0.038**
Role-physical score	34.09 ± 3.90	64.39 ± 3.77	**<0.001** ^b^	37.12 ± 3.94	48.48 ± 3.25	**0.009** ^b^
Bodily pain score	52.91 ± 2.18	76.39 ± 2.72	**<0.001** ^a^	47.00 ± 2.32	65.09 ± 2.42	**<0.001** ^a^
General health score	30.36 ± 1.62	62.21 ± 1.76	**<0.001** ^b^	34.70 ± 1.94	49.76 ± 2.01	**<0.001** ^b^
Vitality score	43.94 ± 1.86	64.55 ± 1.40	**<0.001** ^a^	40.46 ± 1.66	47.43 ± 1.66	**0.011** ^a^
Social functioning score	38.64 ± 1.99	71.21 ± 1.49	**<0.001** ^b^	32.95 ± 2.36	56.06 ± 1.55	**<0.001** ^b^
Role-emotional score	53.54 ± 4.57	76.77 ± 3.40	**0.002** ^b^	48.48 ± 4.61	58.59 ± 4.11	0.085^b^
Mental health score	47.40 ± 1.85	65.58 ± 1.31	**<0.001** ^a^	48.36 ± 1.32	57.33 ± 1.38	**<0.001** ^a^
Health transition score	15.91 ± 3.23	68.94 ± 3.27	**<0.001** ^b^	20.45 ± 3.16	43.18 ± 3.49	**<0.001** ^b^
CD-RISC score	40.00 ± 6.61	65.12 ± 5.21	**<0.001** ^b^	42.03 ± 4.42	50.36 ± 4.70	**<0.001** ^b^

^a^Matched samples *t*-test; ^b^Wilcoxon signed-rank test; *CD-RISC*, Connor- Davidson resilience scale; Significant *p* values are shown in bold type.

### Psychological resilience

**Figure 5:**
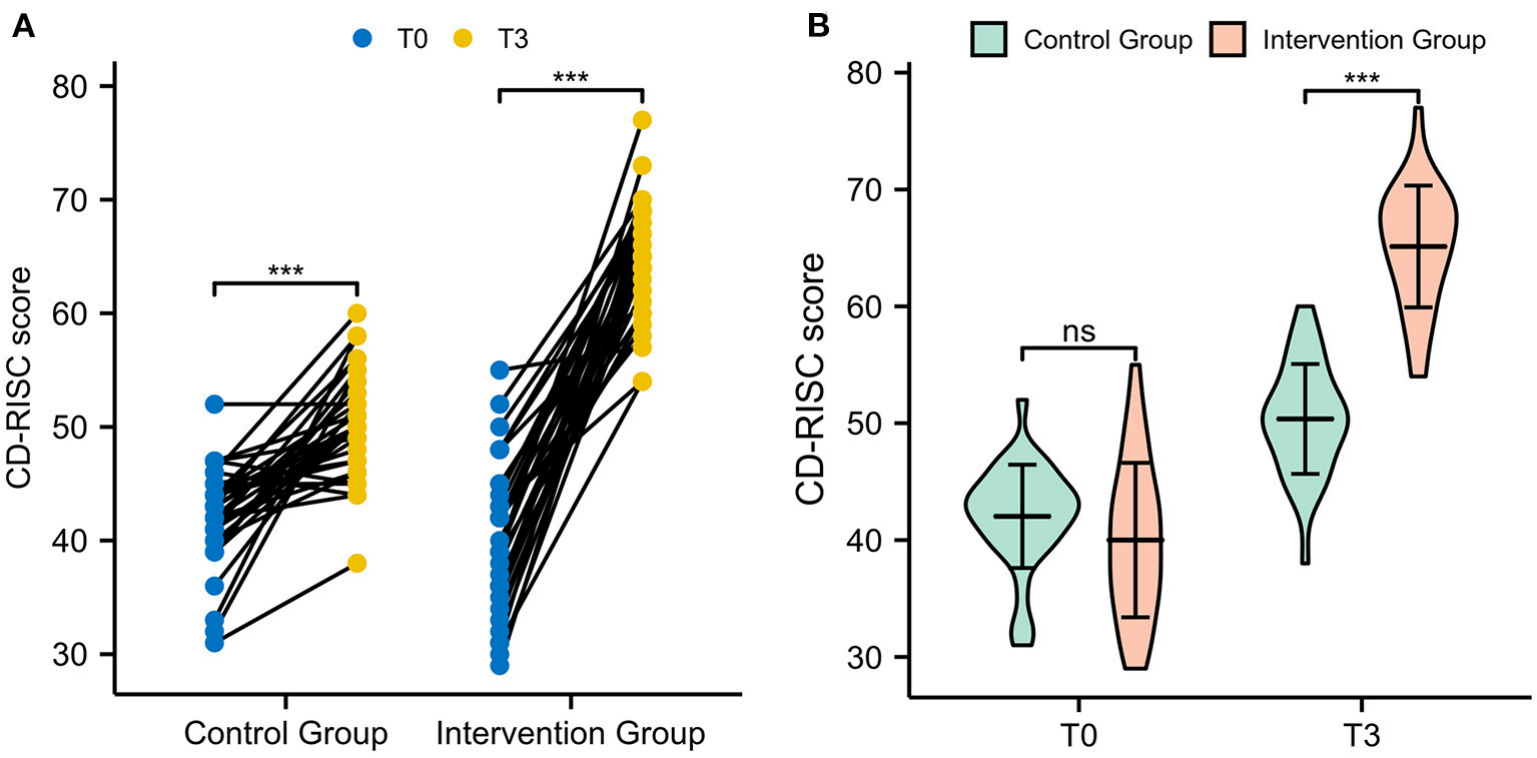
The CD-RISC scores of patients. **(A)** Comparison of CD-RISC scores between T0 and T3 in paired samples. **(B)** Comparison of CD-RISC scores between the intervention group and the control group.

The CD-RISC was used to assess the psychological resilience of patients. As shown in Figure 5A, the CD-RISC scores for the control group were 42.03 ± 4.42 at T0 and 50.36 ± 4.70 at T3. The scores for the intervention group were 40.00 ± 6.61 at T0 and 65.12 ± 5.21 at T3. Both the intervention and control groups demonstrated a significant increase after 3 months (both *p* < 0.001). Furthermore, the CD-RISC score in the intervention group was significantly higher than that in the control group (*p* < 0.001, Figure 5B).

## Discussion

PICC is one of the most common central venous accesses for cancer patients receiving chemotherapy. Although PICC shows unique advantages in clinical application and nursing care, the incidence of complications cannot be ignored (17–19). One of the most frequent and detrimental complications is asymptomatic PICC-RT (20, 21). A meta-analysis of 10 studies comprising 1,591 participants determined that the pooled incidence of asymptomatic PICC-RT in cancer patients was 19% (95% CI, 0.13–0.26) (12). Although asymptomatic PICC-RT does not show severe clinical symptoms, it also has a profoundly negative impact on patients' quality of life. Patients undergoing asymptomatic PICC-RT should establish new lifestyle and diet habits. PICC maintenance and anticoagulant therapy also bring many difficulties and challenges to patients (22). In addition, patients may suffer from discrimination because of the conspicuous presence of devices and dressings on upper limbs (23). Various factors can lead to poor quality of life and great psychological distress in patients. Hence, it is of paramount importance to focus on patients' psychological status and quality of life. In the present study, we investigated the benefits of an online multimodal nursing program on the quality of life and psychological resilience of asymptomatic PICC-RT patients.

In this study, the patients in the intervention group presented higher SF-36 and CD-RISC scores than those in the control group. This verified that the application of this online multimodal nursing program could help asymptomatic PICC-RT patients obtain better health-related quality of life and psychological resilience. The maintenance of PICCs is very complicated; patients cannot always learn thoroughly in the short hospitalization period after PICC placement, and some patients living in remote areas even lack professional medical education. Therefore, asymptomatic PICC-RT patients often show depression and anxiety after discharge (24). Through the online multimodal nursing program, patients can learn the relevant knowledge by themselves at home. Furthermore, the multimodal nursing program regularly provides recommendations on patients' lifestyle and corrects their improper procedures in time. The psychological counselors closely communicate with patients and give them mental persuasion and support. All of this continuing nursing care could effectively alleviate patients' negative emotions and eliminate their worries. In previous reports, the effectiveness of the multimodal nursing program was confirmed in other diseases. Kaina Zhou and colleagues designed a randomized controlled trial and found that the WeChat-based multimodal nursing program could significantly improve health-related quality of life in women with postoperative breast cancer (25). Lingjuan Li et al. indicated that the family participatory nursing model could help lung cancer patients improve their psychological elasticity and quality of life. As a consequence, the multimodal nursing program is worth popularizing and applying in routine nursing care.

Since 2020, coronavirus disease 2019 (COVID-19) has caused extensive economic and social damage worldwide. Many countries offered policies to restrict travel, parties, and outdoor activities of the public to mitigate the spread (26, 27). However, these disruptions significantly impacted the global economy and simultaneously disrupted daily life (28). In addition, the outbreak of COVID-19 and the confinement of the public added pressure on the health care system. Under these circumstances, the development and implementation of telemedicine is urgently needed (29). Thanks to the rapid development and advancement of internet technology, telemedicine has been achieved in diverse medical fields (30, 31). In this research, we established an online multimodal nursing program to help asymptomatic PICC-RT patients. The investigators and psychological counselors could communicate with patients directly at any time. Compared with traditional medical service, this online multimodal nursing program was a success for both nurses and patients. On the one hand, the nurses could follow patients more easily and systematically. On the other hand, the patients could receive help and guidance in time. An additional advantage is that it reduced the risk of transmission. Hence, it is worth developing and utilizing an online multimodal nursing program, especially in the era of COVID-19.

Compared to other studies, the strength of our study is that we included a very specific cohort with asymptomatic PICC-RT. This study was the first to explore the value of an online multimodal nursing program in this type of patient. However, a few limitations cannot be ignored. First, our research was not a double-blinded randomized controlled trial, which might cause diagnostic suspicion bias and reporting bias. Second, a multicenter study with larger sample volumes is needed to assess the benefits of this multimodal nursing program. Third, in the 3-month follow-up period, some potential confounding factors might have impacted the quality of life and psychological resilience. Lastly, the follow-up period in this study was only 3 months. Longer follow-up is required to assess more distant outcomes.

## Conclusion

In conclusion, this study revealed that the application of an online multimodal nursing program could significantly improve the health-related quality of life and psychological resilience of asymptomatic PICC-RT patients. The findings provide evidence to support the necessity of a multimodal nursing program in routine long-term follow-up, especially in the era of COVID-19. However, to establish and popularize an impeccable telemedical program within routine care, the technical and regulatory burdens must be overcome.

## Data availability statement

The raw data supporting the conclusions of this article will be made available by the authors, without undue reservation.

## Ethics statement

The studies involving human participants were reviewed and approved by the Ethics Committee of Guangdong Provincial People's Hospital, Guangdong Academy of Medical Sciences (China). The patients/participants provided their written informed consent to participate in this study.

## Author contributions

All authors contributed to the study conception and design. Study design and project development was performed by XZ. Data collection and analysis was performed by XZ, XH, MX, SZ, YC, and LW. The first draft of the manuscript was written by XH and MX. All authors commented on previous versions of the manuscript. All authors read and approved the final manuscript.

## Funding

This research was funded by Medical Scientific Research Foundation of Guangdong Province, Grant No. A2021350.

## Conflict of interest

The authors declare that the research was conducted in the absence of any commercial or financial relationships that could be construed as a potential conflict of interest.

## Publisher's note

All claims expressed in this article are solely those of the authors and do not necessarily represent those of their affiliated organizations, or those of the publisher, the editors and the reviewers. Any product that may be evaluated in this article, or claim that may be made by its manufacturer, is not guaranteed or endorsed by the publisher.
