# Supplementary material for: Benefits of an online multimodal nursing program among patients with peripherally inserted central catheter-related thrombosis
Source: Front Public Health. 2022 Sep 20;10:971363. doi: 10.3389/fpubh.2022.971363 (PMC9531013; doi:10.3389/fpubh.2022.971363)
Supplement: Supplementary file 2 [file Data_Sheet_2.PDF]

Connor-Davidson Resilience Scale -25

Name: ____________________ Ref. Dr:________________ Date: _______
ID#: ________________ Age: _______

The Connor-Davidson Resilience Scale (CD-RISC-25) is a self-administered scale containing 25 items that exhibit good psychometric properties. Please answer the following 25 questions.

1. I am able to adapt to change.

○ Not true at all. ○ Rarely true. ○ Sometimes true.

○ Often true. ○ True nearly all the time.

2. I have close and secure relationship

○ Not true at all. ○ Rarely true. ○ Sometimes true.

○ Often true. ○ True nearly all the time.

3. Sometimes fate or God helps me.

○ Not true at all. ○ Rarely true. ○ Sometimes true.

○ Often true. ○ True nearly all the time.

4. I can deal with whatever comes my way.

○ Not true at all. ○ Rarely true. ○ Sometimes true.

○ Often true. ○ True nearly all the time.

5. Past successes give me confidence for new challenges.

○ Not true at all. ○ Rarely true. ○ Sometimes true.

○ Often true. ○ True nearly all the time.

6. I try to see the humorous side of things.

○ Not true at all. ○ Rarely true. ○ Sometimes true.

○ Often true. ○ True nearly all the time.

7. Having to cope with stress can make me stronger.

○ Not true at all. ○ Rarely true. ○ Sometimes true.

○ Often true. ○ True nearly all the time.

8. I tend to bounce back after illness, injury or other hardships.

○ Not true at all. ○ Rarely true. ○ Sometimes true.

○ Often true. ○ True nearly all the time.

9. Things happen for a reason

○ Not true at all. ○ Rarely true. ○ Sometimes true.

○ Often true. ○ True nearly all the time.

10. I give my best effort no matter what.

○ Not true at all. ○ Rarely true. ○ Sometimes true.

○ Often true. ○ True nearly all the time.

11. I can achieve my goals.

○ Not true at all. ○ Rarely true. ○ Sometimes true.

○ Often true. ○ True nearly all the time.

12. When things look hopeless, I do not give up.

○ Not true at all. ○ Rarely true. ○ Sometimes true.

○ Often true. ○ True nearly all the time.

13. I know where to turn for help.

○ Not true at all. ○ Rarely true. ○ Sometimes true.

○ Often true. ○ True nearly all the time.

14. Under pressure, I stay focused and think clearly.

○ Not true at all. ○ Rarely true. ○ Sometimes true.

○ Often true. ○ True nearly all the time.

15. I prefer to take the lead in problem-solving.

○ Not true at all. ○ Rarely true. ○ Sometimes true.

○ Often true. ○ True nearly all the time.

16. I am not easily discouraged by failure.

○ Not true at all. ○ Rarely true. ○ Sometimes true.

○ Often true. ○ True nearly all the time.

17. I think of myself as a strong person.

○ Not true at all. ○ Rarely true. ○ Sometimes true.

○ Often true. ○ True nearly all the time.

18. I can make unpopular or difficult decisions.

○ Not true at all. ○ Rarely true. ○ Sometimes true.

○ Often true. ○ True nearly all the time.

19. I can handle unpleasant feelings.

○ Not true at all. ○ Rarely true. ○ Sometimes true.

○ Often true. ○ True nearly all the time.

20. I have to act on a hunch.

○ Not true at all. ○ Rarely true. ○ Sometimes true.

○ Often true. ○ True nearly all the time.

21. I have a strong sense of purpose.

○ Not true at all. ○ Rarely true. ○ Sometimes true.

○ Often true. ○ True nearly all the time.

22. I feel like I am in control.

○ Not true at all. ○ Rarely true. ○ Sometimes true.

○ Often true. ○ True nearly all the time.

23. I like challenges.

○ Not true at all. ○ Rarely true. ○ Sometimes true.

○ Often true. ○ True nearly all the time.

24. I work to attain goals.

○ Not true at all. ○ Rarely true. ○ Sometimes true.

○ Often true. ○ True nearly all the time.

25. I take pride in my achievements.

○ Not true at all. ○ Rarely true. ○ Sometimes true.

○ Often true. ○ True nearly all the time.
